# Discrimination between Modal, Breathy and Pressed Voice for Single Vowels Using Neck-Surface Vibration Signals

**DOI:** 10.3390/app9071505

**Published:** 2019-04-11

**Authors:** Zhengdong Lei, Evan Kennedy, Laura Fasanella, Nicole Yee-Key Li-Jessen, Luc Mongeau

**Affiliations:** 1 Department of Mechanical Engineering, McGill University, Montreal, QC H3A 0G4, Canada;; 2 School of Communication Sciences and Disorders, McGill University, Montreal, QC H3A 0G4, Canada;

**Keywords:** neck-surface vibration, machine learning, voice type discrimination

## Abstract

The purpose of this study was to investigate the feasibility of using neck-surface acceleration signals to discriminate between modal, breathy and pressed voice. Voice data for five English single vowels were collected from 31 female native Canadian English speakers using a portable Neck Surface Accelerometer (NSA) and a condenser microphone. Firstly, auditory-perceptual ratings were conducted by five clinically-certificated Speech Language Pathologists (SLPs) to categorize voice type using the audio recordings. Intra- and inter-rater analyses were used to determine the SLPs’ reliability for the perceptual categorization task. Mixed-type samples were screened out, and congruent samples were kept for the subsequent classification task. Secondly, features such as spectral harmonics, jitter, shimmer and spectral entropy were extracted from the NSA data. Supervised learning algorithms were used to map feature vectors to voice type categories. A feature wrapper strategy was used to evaluate the contribution of each feature or feature combinations to the classification between different voice types. The results showed that the highest classification accuracy on a full set was 82.5%. The breathy voice classification accuracy was notably greater (approximately 12%) than those of the other two voice types. Shimmer and spectral entropy were the best correlated metrics for the classification accuracy.

## 1. Introduction

Voice quality describes a wide range of multifaceted perceptual characteristics of human voice [1]. One of these characteristics is the voice type. Breathy, modal and pressed voice have been viewed on a continuum paradigm of phonation in terms of vocal fold contact area and open quotient [2]. Electroglottalgraph waveforms have shown that breathy voice featured a small Vocal Fold (VF) contact area and a large open quotient, and thus implied a low laryngeal resistance [3]. Pressed voice displayed opposite trends [3]. Methods of voice type classification may broadly be subdivided into two categories: (1) subjective methods; and (2) objective methods. The perceptual judgement of voice quality by panels of listeners (as opposed to self-evaluation) is still considered the gold standard for clinical voice evaluation and monitoring [4–7]. Recency effect and listener experience could confound an individual’s perceptual ratings [8,9]. Notably, as perceptual voice assessment tools are context-specific, clinicians have limited ability to provide long-term monitoring and evaluation of patient’s voice quality outside the clinic. Long-term voice data acquisition and monitoring is known to be challenging to implement in occupational settings. Automatic voice type discrimination based on ambulatory device data is therefore needed.

Objective methods involve the processing of dynamic signals obtained from sensors and the calculation of metrics using a vast array of signal processing schemes. The microphone has primarily been used in a vast majority of voice quality studies [10–13]. Although microphones are convenient, easy to use in the field and have a large bandwidth, the captured voice signals are often contaminated by background noise, and they are distorted by reverberation in the surrounding spaces. The large bandwidth of the microphone provides a broad spectral range, but the associated speech intelligibility implies privacy disclosure, which may be of concern for long-term voice monitoring applications.

The Neck Surface Accelerometer (NSA) offers a viable alternative to microphones for the capture of voice signals [14–18]. The NSA is a small sensor that measures the vibration acceleration in the direction normal to the neck surface. During speech, neck tissue vibrations are mainly produced by the transmission of structure-borne vibrations induced by the acoustic waves in the subglottal and supraglottal portions of the vocal tract to the neck skin surface. Data are acquired through a wearable recorder or a smartphone. The NSA has a good sensitivity and bandwidth, but the neck tissue tends to dissipate high frequency vibrations, and thereby acts as a low-pass filter. Consequently, the bandwidth of the NSA signals is less than 1.5 kHz. The NSA mounted on the below-glottis skin surface can hardly capture formant information, which is important for speech intelligibility. Thus, the NSA protects speakers’ privacy.

The NSA signals accurately convey the features of the voice source such as fundamental frequency (f0) and vocal amplitude [19]. A semi-empirical model was reported to correlate Surface Acceleration Level (SAL) with Sound Pressure Level (SPL) based on 27 participants’ data [20]. Glottal source characteristics such as the Maximum Flow Declination Rate (MFDR) and Harmonic Richness Factor (HRF) have been estimated from NSA signals using subglottal inverse filtering [21]. A comparison between vocal function measures derived from an acoustic microphone and from am NSA has also shown that f0 and jitter are congruent (*p* > 0.98), but shimmer is less correlated (*p* < 0.7) [17].

The first level of objective voice evaluation and classification is normally based on statistical analysis on voice metrics extracted from recorded voice signals. A plethora of temporal, spectral and cepstral metrics have been proposed [2,22–24]. Jitter and shimmer measures of fundamental frequency and loudness perturbations were found to correlate with voice quality based on microphone data [25]. MFCCs and Cepstral Peak Prominence (CPP) have been used to discriminate between modal, breathy, strained and other voice types based on microphone data [11,26]. The difference between the amplitudes of the first two harmonic components on microphone and NSA spectra, *H*1–*H*2, was found to be correlated with perceived speech breathiness [13,14]. Glottal flow waveforms obtained using the Glottal Inverse Filtering (GIF) method have been used for differentiation between modal and pathological voice through the estimation of the MFDR and other residue quantities [27,28]. However, the accuracies of processing close rounded vowels using the GIF method were unacceptable and would limit the use of the GIF for continuous speech. Thus far, no single metric could show a good statistical separation between different voice types, such as modal, breathy and pressed.

A second level of analysis that uses multiple features is usually needed to obtain effective evaluation. Secondary processing tools, such as expert systems or machine learning algorithms, require that the mathematical algorithms first be calibrated through the adjustments of model parameters that are data-specific. The Hidden Markov Model (HMM), Support Vector Machine (SVM) and Artificial Neural Network (NN) were used to improve the performance on classification between different voice qualities based on microphone features [29,30]. A Gaussian Mixture Model (GMM) using Mel-Frequency spectral Coefficients (MFCCs) extracted from neck-surface vibration signals achieved 80.2% and 89.5% accuracies in classifying modal, breathy, pressed and rough types at the frame level and utterance level, respectively [11]. Machine learning algorithms critically require, for accurate results, that the calibration be done against a “ground truth”, or gold standard. In the case of voice quality classification, the “ground truth” consists of subjective evaluation by a panel of trained listeners, with intra- and inter-rater analysis [11].

The NSA technology has recently been applied for voice type discrimination with the use of a Gaussian mixture model [31]. However, the work reported so far in this area has not used a comprehensive listener panel dataset with inter- and intra-rater analysis as the ground truth. The present study attempted to bridge this gap. The overarching idea is to provide automatic detection of voice type and provide long-term monitoring of voice routines based on accumulated datasets. The objective of the present study was to investigate the feasibility of using NSA signals and supervised learning techniques to discriminate between three voice types, namely modal, breathy and pressed. The paper is organized as follows: Section 2 describes the NSA hardware and the data acquisition process. Section 3 describes the subjective auditory-perceptual voice type rating task using an online system. Section 4 describes how features were extracted and used for machine learning classification. Section 5 shows the classification and analysis results based on different feature sets. The conclusions, the limitations of this study and future work are discussed in Section 6.

## 2. Experimental Setup and Data Acquisition

### 2.1. Hardware Platform Description

**Figure 1.**
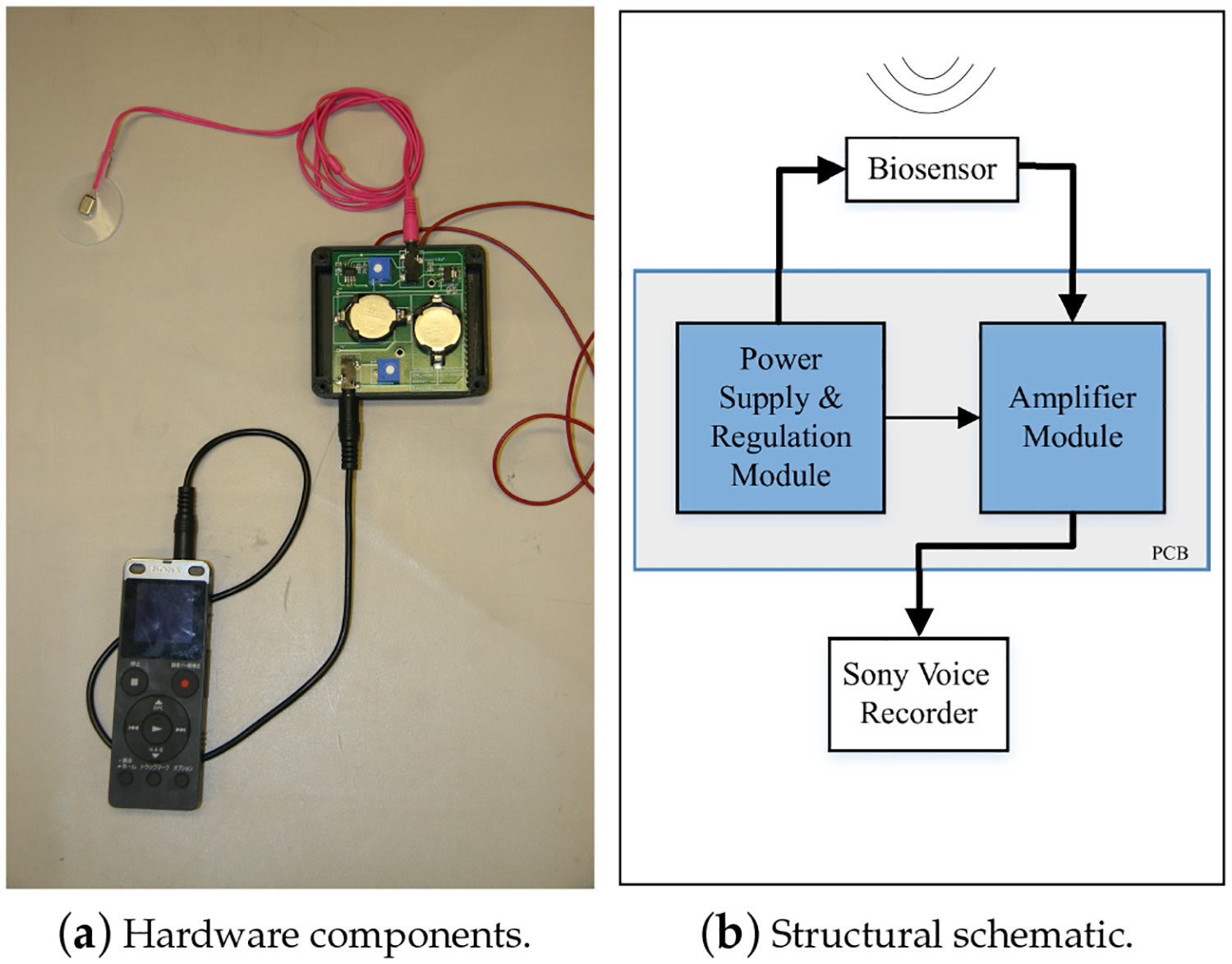
The physical prototype and schematic design of the NSA.

**Figure 2.**
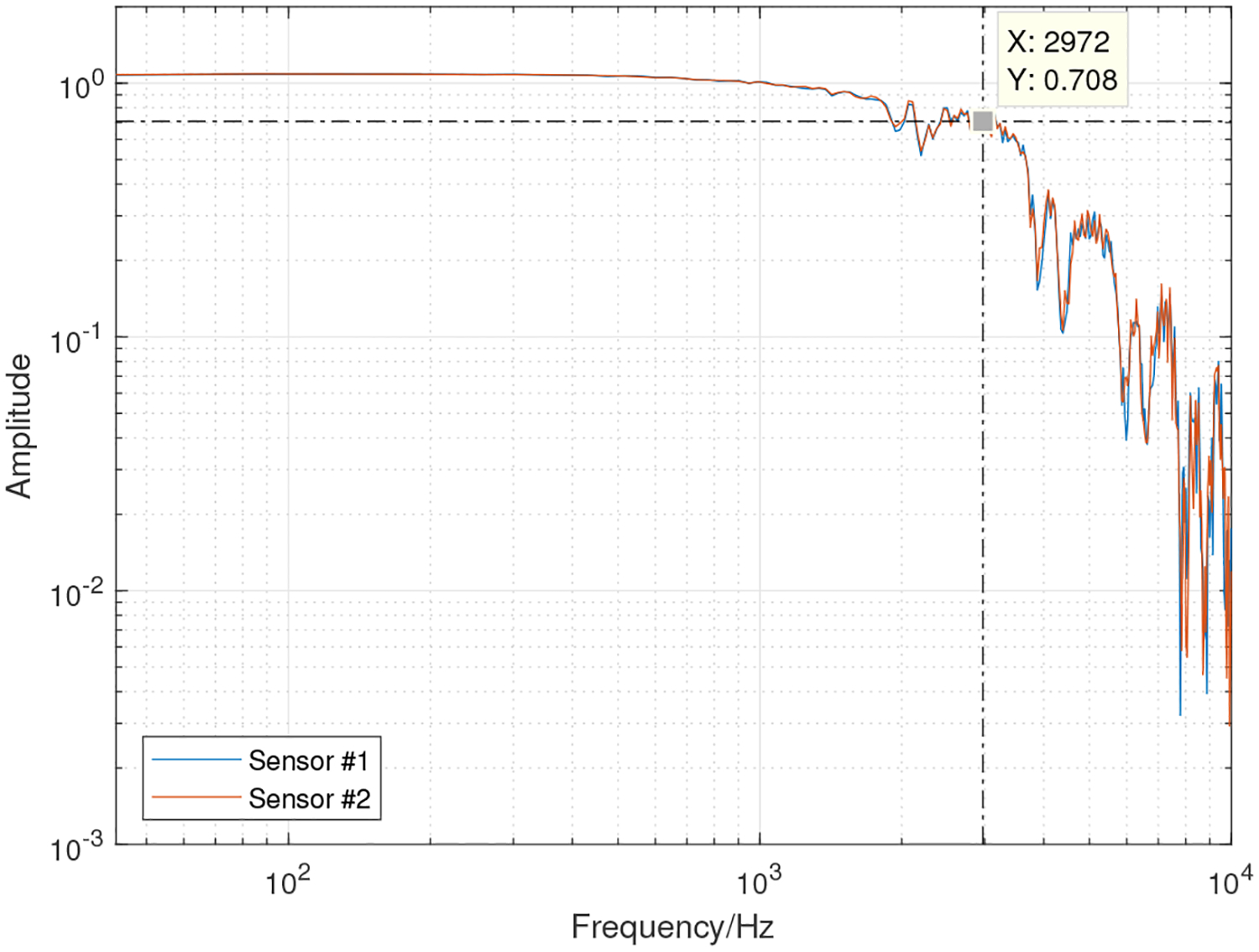
The transfer function between the LDV and the NSA signals for two sensors.

The acoustic sensor used in the NSA for neck-skin vibration monitoring was a miniature accelerometer (BU-27135, Knowles Inc., Itasca, IL, USA). One customized peripheral circuit, consisting of one power supply module and one amplifier module, was fabricated using the Printed Circuit Board (PCB) technique. Four lithium coin batteries (CR2032, Panasonic Inc., Japan; nominal voltage and capacity: 3 V and 225 mAh) were used as the power source. The interface between the peripheral circuit board and the accelerometer was one 3.5-mm stereo audio cable, of which the three wires corresponded to the positive, ground and signal channels. One voice recorder (ICD-UX565F, Sony Inc., Tokyo, Japan) was employed to record the acceleration data with high fidelity. The hardware components are shown in Figure 1. To increase the sensitivity of the accelerometer, one silicon pad (Ecoflex 00–30, Part A and Part B with a 1:1 ratio, Smooth-On Inc., Macungie, PA, USA) was fabricated to encapsulate the accelerometer and increase the contact area between the accelerometer and neck skin. The silicon pad was moulded to obtain a flat (diameter: 28 cm) and thin (thickness: 1.2 mm) circular pedestal. An adhesive was used to mount the silicon pad on neck skin firmly. The Sony voice recorder supports multiple options for sampling rate, encoding format (MP3: 48 kbps/128 kpbs/192 kbps; Linear Pulse Code Modulation (LPCM): 44.1 kHz, 16-bit) and compatible memory cards. The voice data were saved in WAV format audio files and were transferred to a PC for analysis through the USB interface. The uniformity of the NSA frequency response was verified using Laser Doppler Velocimetry (LDV) in a Gaussian white noise test. The transfer function between the LDV and the NSA signals is shown in Figure 2. The frequency bandwidth of the NSA was around 3000 Hz.

### 2.2. Voice Recording Process

The human study protocol (A09-M46–11A) was approved by the Institutional Review Board at McGill University. The experiment was conducted in a voice recording studio. Participants were 31 native Canadian English speakers aged from 18–40 years. All participants were females and had no history of voice disorders or laryngeal discomfort during the experiment, as self-reported subjectively. The protocol for each participant began with a training session followed by a formal recording session. The duration of the entire session for each participant was approximately 30 min. During the training session, the participants were instructed by an SLP to practice producing five vowels in the three target voice types (modal, breathy and pressed). The five vowels were [aː] in “father’, [æ] in “cat”, [e] in “bed”, [iː] in “heat” and [uː] in “food”. Each vowel is characterized by one unique vocal tract shape [32]. In this experiment, only glottal articulators (i.e., intrinsic laryngeal muscles) were expected to vary across different voice qualities. The vocal tract shape was presumed to remain constant for the same vowel. The influence of pitch and loudness on voice type discrimination was not considered and was left outside the scope of this study. The SLP provided exemplars of different voice types and kinesthetic tasks of laryngeal muscles to aid accurate replication of modal, breathy and pressed voice types. The participants did not proceed to formal recording until the SLP confirmed they could reliably produce all three voice types. During the formal recording session, the participants were required to pronounce stable and sustained vowels for at least two seconds. The Linear Pulse Code Modulation (LPCM) encoding mode with a 44.1-kHz sampling rate in the SONY voice recorder was used. The participants were required to produce the three different types with short breaks between each vowel. During each utterance, the participants were required to maintain the pitch and loudness at their greatest effort. Repetition of the corresponding utterance was required until the target voice type was met, as judged by the SLP.

## 3. Auditory-Perceptual Screening

**Figure 3.**
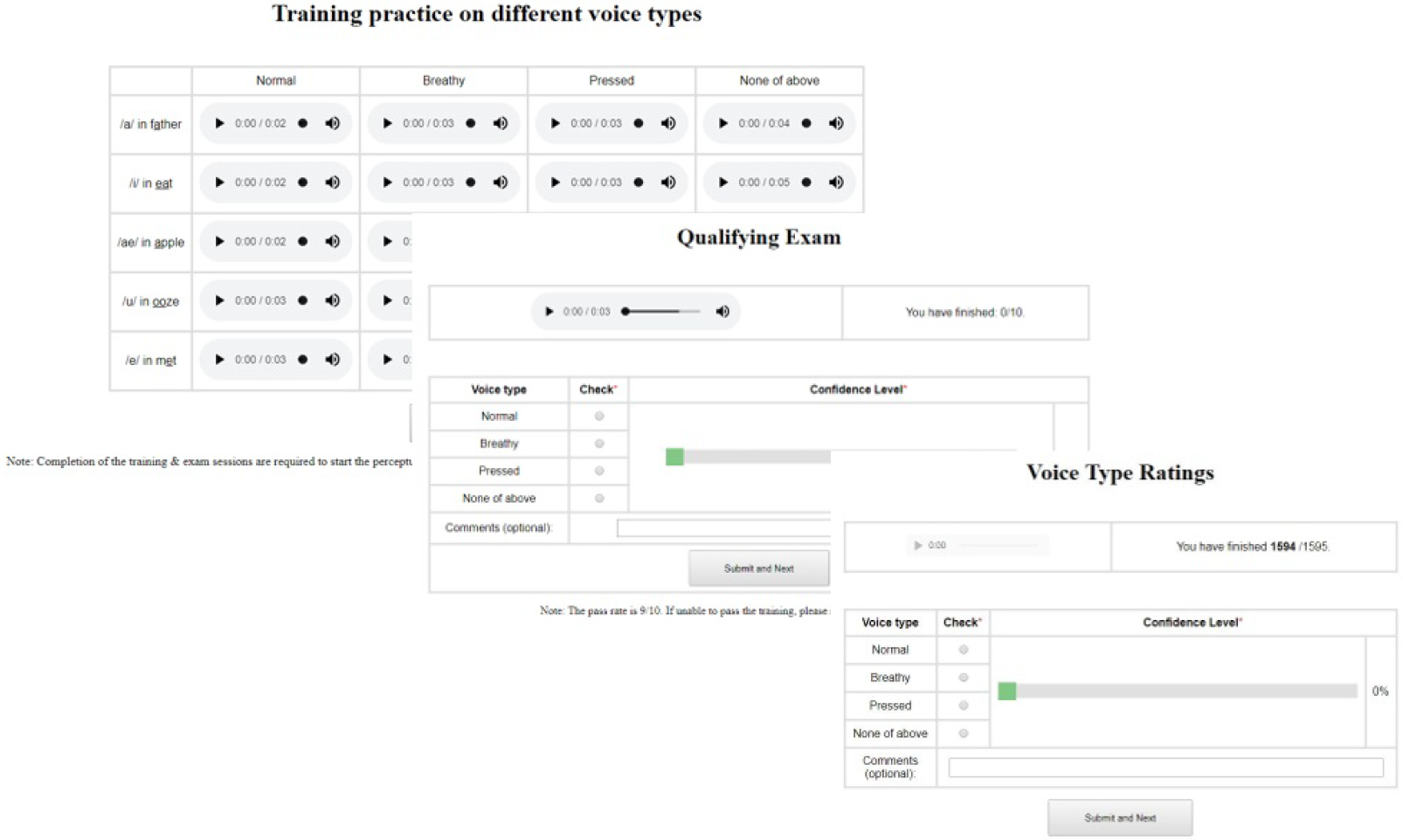
Screenshots of the online voice type assessment system.

Most participants experienced difficulties in producing consistent breathy and pressed voice types, as reported by the on-site monitoring SLP. A mixed voice quality or other quality dimensions such as vocal tremor were sometimes perceived in the formal recording session. The recorded microphone data were perceptually assessed independently by five SLPs in order to validate each token’s membership to a voice quality category. The SLPs were blinded to the purpose of the study and the information of voice samples. Since there is no validated perceptual tool to facilitate assessment of voice quality in two-second tokens, a customized website was developed to allow the SLPs to categorize the single vowel samples. Screenshots of the online system are shown in Figure 3. The SLPs were required to select a voice quality category (modal, breathy, pressed or none of the above) and a confidence level for the specific token’s categorical membership. Distractor samples, with other voice quality dimensions, such as tremor, were added into the dataset to ensure use of the “none of the above” category and reduce any selection bias towards the three target types. The comment box was utilized to describe perceptually any samples categorized as “none of above”. The SLPs were required to pass a training test (eight of 10 correctly categorized sample tokens) before proceeding to the formal rating.

Inter-rater reliability analysis was used to evaluate the agreement among raters. Fleiss’ Kappa method was used to assess the SLPs’ reliabilities on categorical ratings. The parameters for the analysis were five raters, four categories (modal, breathy, pressed and none of the above) and 1595 samples. According to the *κ* value interpretation proposed by Landis and Koch, the resulting (*κ* = 0.7322) value indicated a substantial reliability among SLPs. Intra-rater reliability was evaluated by calculating the ICC (Intra-class Correlation Coefficient) using IBM SPSS Statistics 24 on 200 duplicated samples. The ICC was calculated using the two-way random model, as participants and raters were chosen randomly from a large population. Absolute agreement was targeted. The *κ* values for all five SLPs revealed “almost perfect” reliability (0.802, 0.863, 0.874, 0.931, 0.952) based on their rating results on the 200 duplicated samples, as interpreted in [33].

The auditory-perceptual screening process was used to obtain ground-truth voice type labels for single vowel phonation and to screen out samples with mixed or inconsistent voice qualities [11]. In this study, “pure samples” were defined as any sample whereupon at least four out of five raters agreed on the voice type label with a minimum confidence level of 80%. The screening results showed that 952 out of 1395 samples were rated as “pure”. The numbers of samples rated as “pure” voice types were 285, 395 and 273, for modal, breathy and pressed voice types, respectively. The number of breathy voice samples was approximately 40% greater than that of each other voice type sample, indicating that breathy voice was easier to mimic and perceive than modal or pressed voice. Through auditory-perceptual screening, the labels of the “pure” samples provided a ground truth dataset for developing an automatic voice type classification algorithm.

## 4. Classification

The NSA and microphone data were measured simultaneously during the experiment and transferred to a desktop computer for data analysis. Labels of “pure” NSA samples were obtained by matching with the “pure” labels obtained from the auditory-perceptual screening. Seven features including *H*_1_, *H*_2_, *H*_3_, *H*_4_, jitter, shimmer and Spectral Entropy (SE) were extracted from the “pure” NSA spectra and time-domain waveforms. Since the loudness was not constrained during the formal data recording session, the amplitudes of the time-domain waveforms and the spectral harmonics were both normalized to eliminate the influence of loudness on the subsequent classification.

### 4.1. Feature Extraction

**Figure 4.**
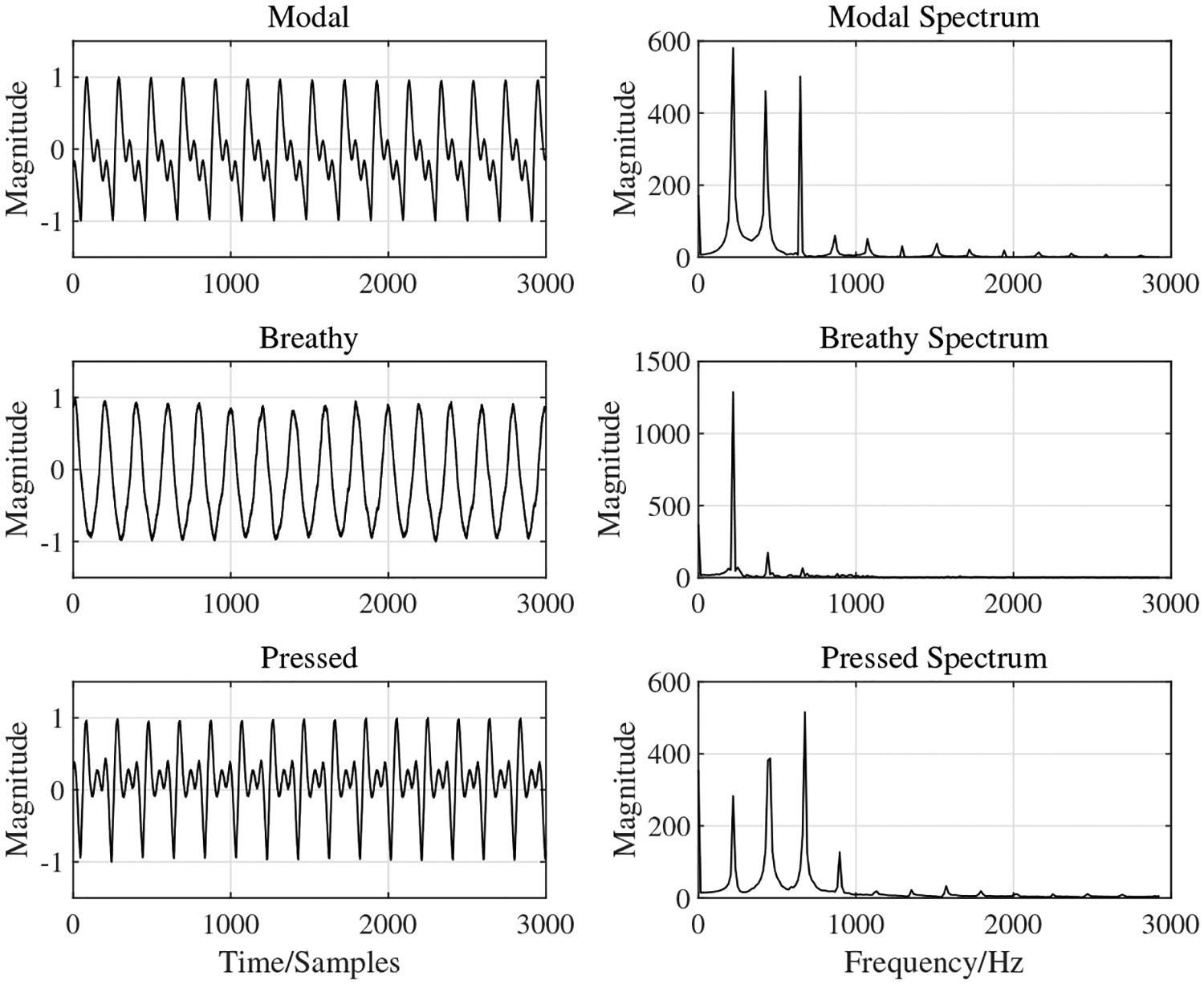
Representative example of normalized NSA waveforms and corresponding spectra of /a:/ in terms of different voice types: modal, breathy and pressed. The frequency resolution is 1 Hz.

**Figure 5.**
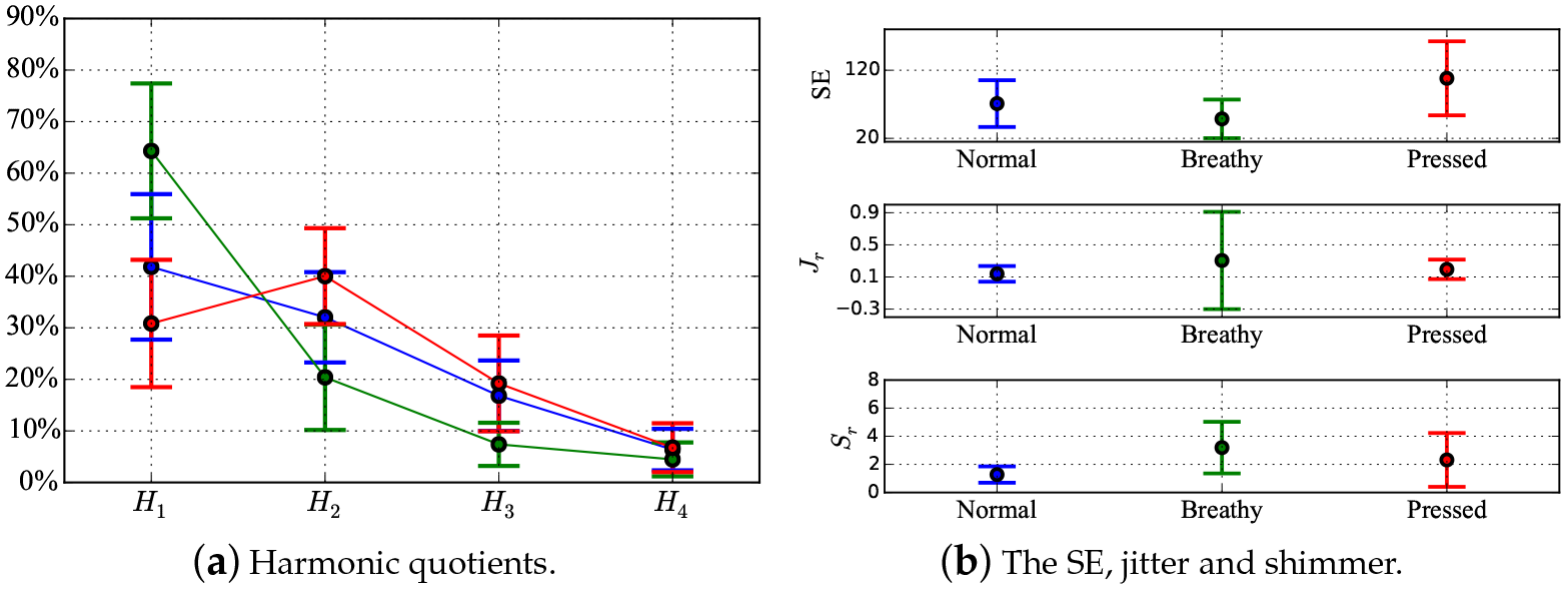
The averages and standard deviations of *H*_1_–*H*_4_, SE, jitter and shimmer based on 31 participants’ “pure” NSA samples. The blue, red and green colours represent the modal, pressed and breathy voice types, respectively.

**Figure 6.**
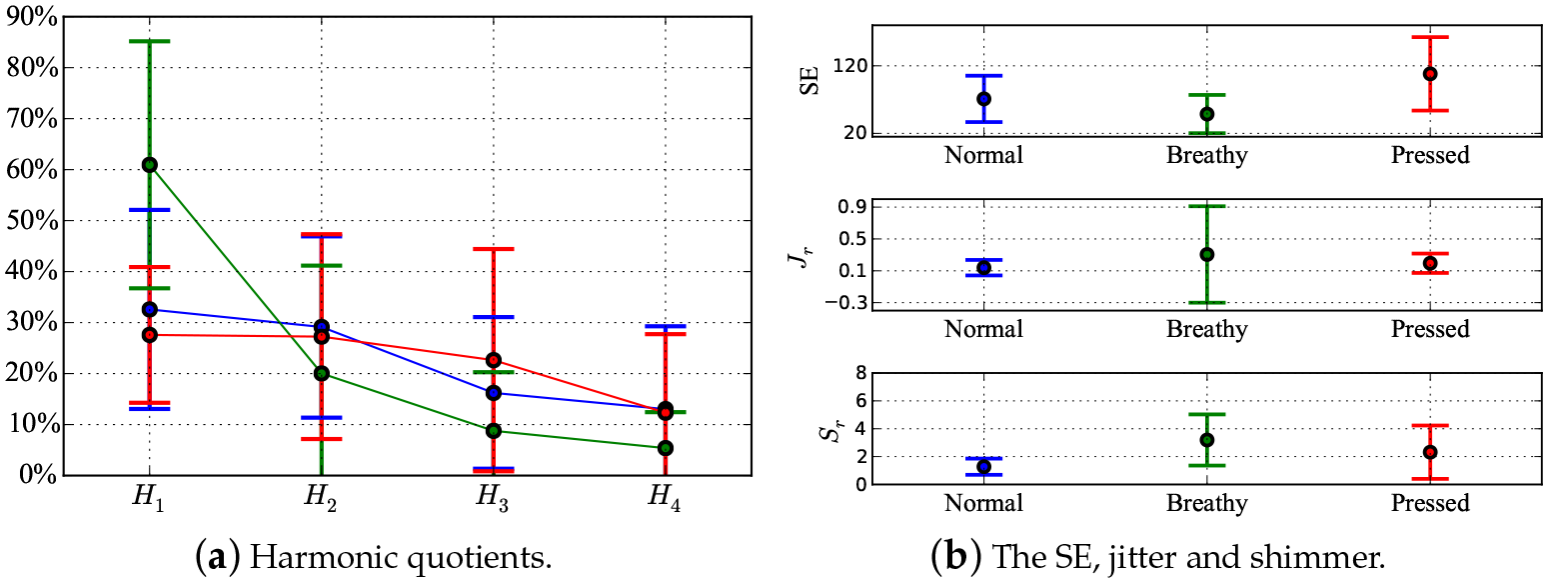
The averages and standard deviations of *H*_1_–*H*_4_, SE, jitter and shimmer based on 14 participants’ “pure” NSA samples for the pilot study. The blue, red and green colours represent the modal, pressed and breathy voice types, respectively.

Features were extracted at the utterance level. The utterances were isolated from the raw data using a Vocal Activity Detection (VAD) algorithm, which was based on the short-time energy and zero crossing rate methods [34]. A Hamming window was used to obtain NSA spectra. The NSA spectral harmonic quotients were calculated using,

(1) $$H_{i}=\frac{A_{i}}{A_{1}+A_{2}+A_{3}+A_{4}+A_{5}}\;(i=1,2,3,4),$$

where *A*_*i*_(*i* = 1, 2, 3, 4, 5) are the amplitudes of the first five harmonics in the NSA spectra. The sum of *H*_*i*_(*i* = 1, 2, 3, 4, 5) is unity according to Equation (1). The first four components, *H*_*i*_(*i* = 1, 2, 3, 4), were selected as features, with *H*_5_ excluded because *H*_5_ was linearly related to *H*_*i*_(*i* = 1, 2, 3, 4) and thus was redundant. The harmonics were used to approximate the envelopes of NSA spectra in a limited frequency bandwidth from 50 Hz–1500 Hz. The SE described the complexity of a signal and was defined as,

(2) $$\text{SE}=-\overset{n}{\underset{i=1}{\sum }}p_{i}\text{log }p_{i},$$

where *p*_*i*_ is the normalized spectral density point, which is from 0–3000 Hz. For a simple signal, e.g., an ideal sinusoidal function, most of the spectral energy was concentrated within a narrow bandwidth, resulting in a small SE. On the contrary, a white noise has a broadly distributed spectrum, and the resulting SE was large. To include voice stability information in the feature set, the jitter and the shimmer were extracted from the time-domain waveforms. Since the lengths of the utterance samples were not identical, jitter (*J*_*r*_) and shimmer (*S*_*r*_) were calculated as relative percentages using,

(3) $$J_{r}=\frac{\frac{1}{N-1}\overset{N-1}{\underset{i=1}{\sum }}\left|T_{i}-T_{i+1}\right|}{\frac{1}{N}\overset{N}{\underset{i=1}{\sum }}T_{i}},$$

and:

(4) $$S_{r}=\frac{\frac{1}{N-1}\overset{N-1}{\underset{i=1}{\sum }}\left|A_{i}-A_{i+1}\right|}{\frac{1}{N}\overset{N}{\underset{i=1}{\sum }}A_{i}}.$$

where *T*_*i*_ and *A*_*i*_(*i* = 1, 2, …*N*) were the period of each vocal cycle and the peak magnitude in each vocal cycle, respectively. *N* was the number of sampling points for each voice sample. The feature vector had the form of [*H*_1_, *H*_2_, *H*_3_, *H*_4_, SE, *J*_*r*_, *S*_*r*_]. One representative NSA waveform and its spectrum are shown in Figure 4. The feature statistics for 31 participants are shown in Figure 5a,b. Figure 5a shows the shapes of the spectral envelopes for each voice type. The breathy voice features a prominent *H*_1_ component, which accounts for over 60% of the total energy. The pressed voice features a prominent *H*_2_. The modal voice harmonics decreased monotonically with frequency from *H*_1_–*H*_4_, but less rapidly than those of the breathy voice. The SE of pressed voice was notably different from that of the other voice types, as shown in Figure 5b. The average SE value of pressed voice was greater, which indicated that the pressed voice spectrum had a greater bandwidth than the other voice types. A comparison between the shimmer values across voice types showed that the modal voice had the smallest average value and standard deviation, which indicated that the modal voice has the most stable loudness. Figure 5a,b shows the notable feature range overlap across voice types, which caused difficulties in voice type classification. The classification results obtained using the Linear Discriminant (LD) method based on single features are shown in Table 1. The overall accuracies for every single feature classification did not exceed 65%. *H*_1_, SE and shimmer contributed the most to the classification accuracies for pressed (from TP to PP: 68%), breathy (from TB to PB: 93%) and modal (from TM to PM: 92%) types, according to the confusion matrix in Table 1. The modal and breathy voice had similar SE distributions. The LD method could not recognize modal voice and misclassified all modal voice samples into breathy or pressed types. The similar jitter distribution across different voice types resulted in a misclassification of all pressed voice samples into modal or breathy voice types. A pilot study was conducted using the same protocol as that of the present study. In the pilot study, participants were 14 female Canadian English speakers aged from 18–40 years with no history of voice disorders. Voice data were recorded using the NSA only. No auditory-perceptual screening was done due to the absence of microphone recordings. Since the sample size (n = 180) of the pilot study was small, the classification of the NSA dataset using machine learning techniques was unsupported. The results of the statistical analysis on various features are shown in Figure 6a,b. Figure 6a shows that the spectral envelopes of the breathy and modal voice of the pilot study had similar contours as those of the present study. The *H*_2_ of the pressed voice of the pilot study was less prominent (12%) than that of the present study. In Figure 6b, the SE and shimmer analysis showed similar trends as those in Figure 5b. The modal voice featured the lowest average value and standard deviation for shimmer, and pressed voice featured the highest average value for the SE. For jitter, the breathy voice of the pilot study showed a larger standard deviation (approximately 0.4 more) than that of the present study. No notable difference between jitter in modal and pressed voice was found between the pilot and the present study. Overall, the results from the pilot study were consistent with those of the more exhaustive present study.

### 4.2. Machine Learning Classification Using Multiple Features

Supervised learning algorithms were implemented using MATLAB (2018a). Our preliminary analysis on classification used more than 10 classifiers and their derivatives based on linear and nonlinear kernel functions. Considering overall classification accuracies and physical interpretation, results were obtained for five classifiers: Linear Discriminant (LD), medium Decision Tree (DT), linear Support Vector Machine (SVM), weighted K-Nearest Neighbours (KNN) and Neural Network (NN). The LD is a simple classifier that uses a linear combination of selected features to test whether the dataset is linearly separable. Since LD does not consider nonlinear metrics, LD directly separated the dataset using feature amplitude range. The medium DT used up to 20 splits and Gini’s diversity index as the split criterion. Our previous analysis showed that the overall classification accuracy was notably lower if the number of splits was smaller than 10 or larger than 40. Linear kernel functions were used to build the SVMs. The linear SVMs used a one-versus-one strategy for multiclass classification and chose the label that was selected by the most linear SVM classifiers. The weighted KNN used 10 nearest neighbours to determine the host’s label. The number of neighbours was optimized in previous pilot studies. The Euclidean distance used by the weighted KNN described the degree of sample aggregation. The decision boundaries were physically meaningful in terms of clustering tightness. The NN used 100 neurons in one hidden layer and the scaled conjugate gradient back-propagation method in updating parameters. Different neuron numbers were tested before, and the number of 100 neurons was shown to have a good classification performance in terms of classification accuracy and computation time. The performance of the NN was evaluated using the cross-entropy method. A five-fold cross-validation was done for all classifiers, with the exception of the NN, to prevent model overfitting. The average accuracy was determined. For the NN classifier, the dataset was randomly divided into three subsets: training (n = 70%), validation (n = 15%) and testing (n = 15%). The data randomization was repeated six times for the NN classification, and the average accuracy was calculated for presentation. Classification was performed on the full feature set and its nine subsets to investigate the priority and the contribution of each feature to the overall accuracies. A leave-one-feature-out (LOFO) strategy was used to build the first seven feature subsets. The *H*_1–4_ + SE subset was a composite of spectral features. The *J*_*r*_ + *S*_*r*_ subset was built using two vocal stability metrics.

## 5. Results and Discussion

The classification results were analysed in multiple dimensions. Firstly, a comparison between the full feature set and the LOFO subsets was made to investigate the contribution of each specific feature to the overall and the individual-class classification accuracies. The accuracy of different classifiers was assessed. Secondly, a comparison between the spectral feature set (*H*_1–4_ + SE) and the stability feature set (*J*_*r*_ + *S*_*r*_) was made to show their contributions to the overall classification accuracies.

### 5.1. Full Set versus LOFO Subsets

**Figure 7.**
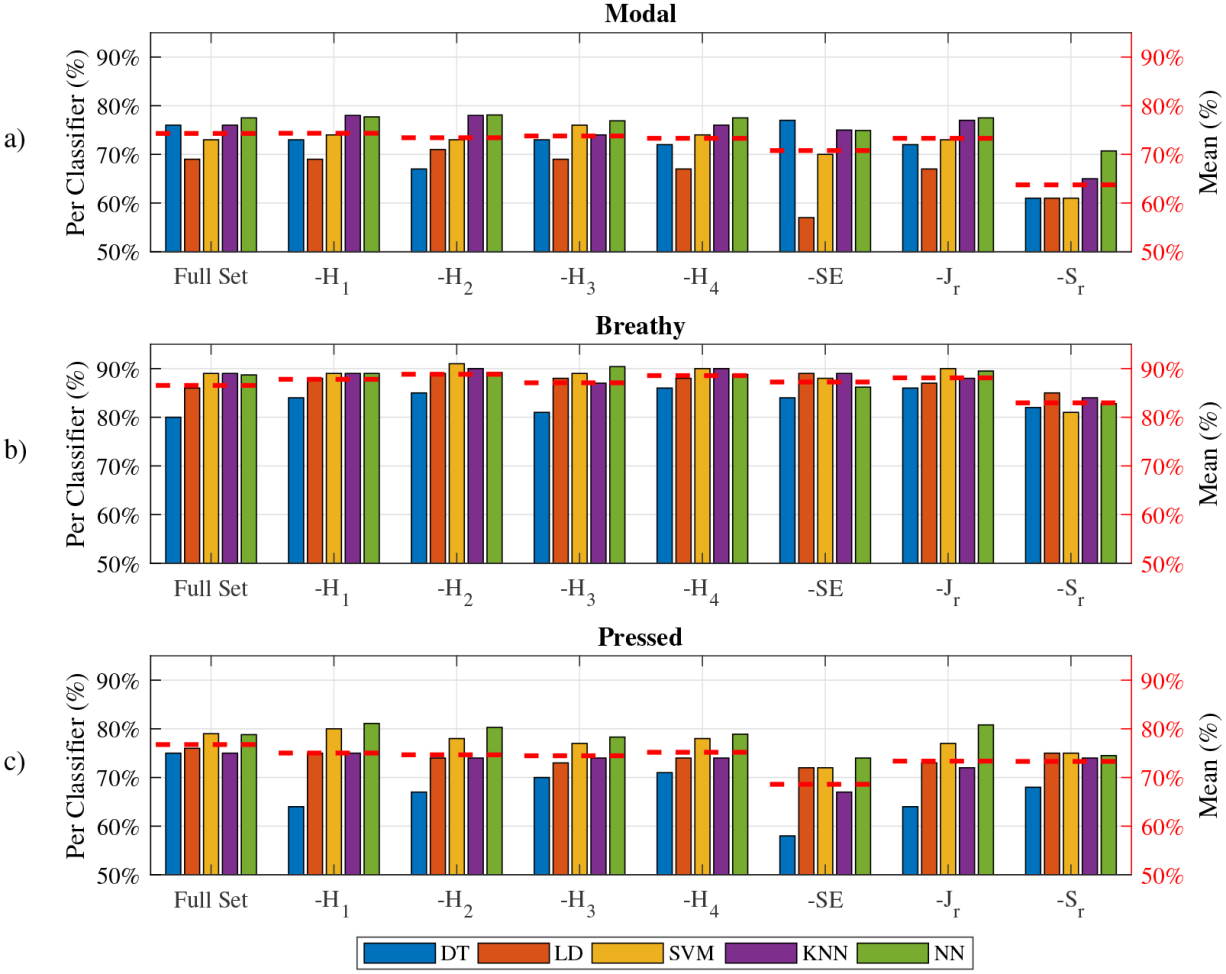
Per-type classification accuracies for the full set and the LOFO subsets. The red dashed line represents an averaged accuracy on a data-set as a function of the classifier. (**a**) Modal voice classification, (**b**) breathy voice classification and (**c**) pressed voice classification.

The overall classification accuracies using the full set and the LOFO subsets are shown in Table 2, and accuracies per voice type classification are shown in Figure 7. Table 2 shows that all classifiers achieved an overall accuracy greater than 75% on the full set. NN (82.5%), SVM (81.3%) and KNN (81.0%) yielded over 80% overall accuracy on the full set, which was much greater (by approximately 20%) than the single feature classification accuracy in Table 1. By removing features from the full set, the overall accuracies were generally decreased with respect to the per-set average of the overall accuracies. Two LOFO subsets, −*S*_*r*_ (74.4%) and -SE (76.7%) dropped by 5.7% and 3.4%, respectively, in comparison with the full set. For these two LOFO subsets, all classifiers showed a notable decrease between −2.5% (KNN on -SE) and 8.1% (SVM on −*S*_*r*_) in overall accuracy. This showed that the shimmer and the SE were of more importance than other features in the classification overall accuracy. Other subsets did not produce conspicuous changes in overall accuracies. Only DT’s performance on −*H*_1_ (−2.5%), −*H*_2_ (−4.0%), −*H*_3_ (−2.0%) and −*J*_*r*_ (2.0%) and LD’s performance on −*J*_*r*_ (−1.5%) were degraded compared to the full set. Other classifiers’ accuracies did not change notably (within ±1.1%). The overall accuracy varied with the chosen classifier. In general, NN, SVM and KNN had greater overall accuracies than DT and LD on both the full set and the LOFO subsets. All accuracies greater than 80% were achieved by these three classifiers. The DT and the LD accuracies did not exceed 80%, either on the full set or the LOFO subsets. For the per-classifier average accuracies, shown in Table 2, the NN achieved the highest overall accuracy (81.5%) on all datasets, and DT had the lowest overall accuracy (75.2%). The classifier performances based on the full feature set were shown in Table 3. The AUC scores for all classifiers exceeded 0.85, which showed satisfactory performances of the classifiers. The NN achieved the highest AUC score (0.93, 0.97), the highest TPR (0.81, 0.90) and the lowest FPR (0.10, 0.08) for the modal and the breathy voice classification. For the pressed voice, the NN AUC score and the TPR score were the highest (0.94) of all classifiers, but the NN FPR (0.16) was not the lowest.

Two Chi-square tests were conducted to evaluate the classification accuracies of classifiers for the full set and the LOFO sets, respectively. Alpha values were set as 0.05. The null hypothesis of the first test was that the classification accuracy was independent of the classifier for the full feature set. Results showed that classification accuracies were significantly different between classifiers (*p* = 0.0268). Furthermore, the NN had the best classification accuracy overall for the full feature set of data. The null hypothesis of the second test was that the classification accuracy was independent of the features selected for classification. The classification accuracies of different datasets were significant for SVM (*p* = 1.99^−6^), KNN (*p* = 5.7 × 10^−3^), NN (*p* = 8.7 × 10^−5^), but insignificant for DT (*p* = 0.0995) and LD (*p* = 0.119). Results suggested that the SVM, KNN and NN classifiers were more sensitive to the variation of the feature set than the other two classifiers (DT and LD).

Figure 7 shows more details on how classifiers and single features influenced the per-type classification accuracies. The results are shown in comparison with the full set of classification results. For the modal voice classification, the −*S*_*r*_ subset results indicated a notable decrease in average accuracy (drawn in the red dashed line). The decrease for all classifiers was 15%, 8%, 12%, 11% and 7%, respectively. The average accuracy of the −*S*_*r*_ was 63.7%, i.e., 10.6% lower than that (74.3%) of the full set. The average accuracy of -SE was also lower (−3.5%) than that of the full set due to the notable degradation (−12%) of the LD performance. Other classifiers’ performance yielded little change (within ±2%) on the -SE subset. The modal voice classification accuracies were comparable between the full set and the other subsets. This result indicated that the other features were less important than SE and the shimmer in the modal voice classification task. NN and the KNN classifier had a better performance (approximately 5% greater) than the other three classifiers on all datasets.

For the breathy voice classification, the accuracies of all datasets using different classifiers were approximately 12% greater than those of the modal and the pressed voice classification. All datasets, except the −*S*_*r*_ set, had very high accuracies (approximately 88%). The −*S*_*r*_ set had a lower average accuracy (approximately 5% lower) than the other datasets, which indicated that the shimmer was important for the breathy voice classification. Other features were less important than shimmer, as no obvious accuracy drops for other LOFO subsets were found. The DT performance was approximately 5% lower than that of the other classifiers. Other classifiers had a comparable performance for the breathy voice classification.

For the pressed voice classification, the SE feature was salient. The -SE average accuracy was 8.1% lower than the full set, and at least 4.8% lower than the other LOFO subsets. All classifiers showed a decreased accuracy on the -SE subset. The −*J*_*r*_ and the −*S*_*r*_ subsets also had decreased accuracies for pressed voice classification, but less notably than the -SE subset. NN had the best accuracy performance between 74% and 81.1% on all datasets. DT had an unsatisfactory accuracy performance between 58% and 71% on the LOFO subsets. Compared with the full set, DT accuracies decreased between −4% and −17%, more than that for the other classifiers on the LOFO subsets.

The LOFO strategy showed that the shimmer and the SE were the most important features for the voice type discrimination task. In particular, shimmer contributed most to the modal voice classification, as observed in Figure 5b. The modal voice samples had a much better aggregation and a lower average value than the other two types for the shimmer feature. The SE contributed most to the pressed voice classification, which could be explained by Figure 5b. The pressed samples featured less overlap with other voice type samples in terms of SE. The breathy voice was much easier to classify using the algorithms than the other two voice types, as shown in Figure 5b.

### 5.2. Full Set versus Spectral Set versus Stability Set

**Figure 8.**
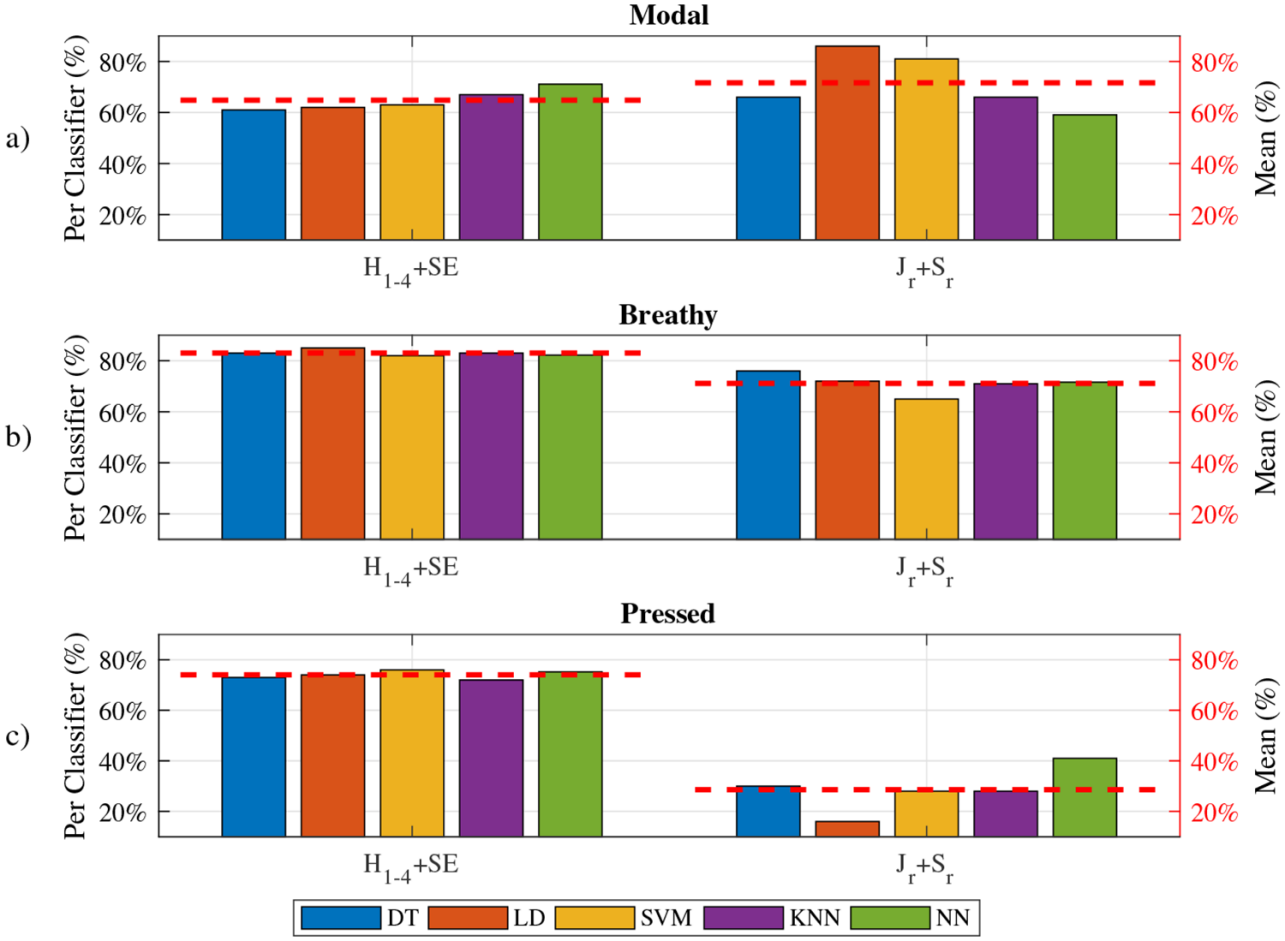
Per-type classification accuracies for the spectral set and the stability set. The red dashed line represents that averaged accuracy on a dataset using different classifiers.

The classification results for the spectral set (*H*_1–4_ + SE) and the stability set (*J*_*r*_ + *S*_*r*_) are shown in Table 4 and Figure 8. Compared with the full set (80.1%), the spectral set achieved a lower, but still satisfactory overall accuracy (75.1%), while the stability set performance was unsatisfactory, as its maximal accuracy was only 61.5%. The addition of stability features into the spectral set for classification yielded an observable improvement of 5% in overall accuracy. The overall accuracies of the spectral set were much greater (15.6% on average) than those of the stability set. The breathy and the pressed voice classification in Figure 8 show that the spectral set yielded greater accuracies than the stability set for all classifiers. The pressed voice was totally indistinguishable using the stability set, as the average accuracy was below 30%. However, the modal voice classification results showed that the stability set had a greater average accuracy (6.8% greater) than the spectral set. This means that the modal voice was more steady than the other two voice types in vocal intensity and fundamental frequency. The LD achieved a very high accuracy (86.0%) for the modal voice classification using the stability set. For breathy and pressed voice classification, all five classifiers achieved a comparable accuracy (83.0% ± 1.2%, 74.0% ± 1.6%) using the spectral set. However, the similar distribution between the breathy and the pressed samples in jitter and shimmer indicated a high possibility of misclassification between these two types using the stability set in Figure 5b.

## 6. Conclusions

In the present study, seven NSA features showed different trends for different voice types. The spectral envelopes of the NSA signals were notably different for each voice type. The breathy voice spectral envelope decreased sharply from *H*_1_–*H*_4_. The modal voice spectral envelope also decreased from *H*_1_–*H*_4_, but less sharply than that of the breathy voice. The pressed voice spectral envelope increased from *H*_1_–*H*_2_ before decreasing from *H*_2_–*H*_4_. The SE revealed a notable difference between the pressed voice type and the other two types. The pressed voice had a much greater average SE (approximately 90% greater) than the other two types of voice. No notable difference between different voice types was found in jitter. The shimmer was the lowest for modal voice, which also had the smallest aggregation. The use of machine learning methods improved the voice type classification accuracies by approximately 20% from 60%–80% compared with the single-feature classification method. In particular, the pressed voice classification accuracy was improved by 13.1%. *S*_*r*_ and SE were found to make the greatest contribution to the machine learning classification. Increased sample size and auditory perceptual screening contributed to reducing the standard deviation of features and improved the differentiation accuracy between modal and pressed voice types.

The main contribution of this study was the successful application of machine learning algorithms to the NSA data for voice type classification. An overall classification accuracy of 82.5% was achieved. The approach proposed here is based on a noninvasive and portable measurement tool, i.e., the NSA, which offers long-term voice data recording capability and speaker privacy preservation. This work may lead to clinical applications. It could assist in the measurement of vocal fatigue and determine voice safety limits by incorporating the voice type factor into the vocal dose measures [35]. Considering that the existing vocal dose metrics cannot perfectly represent the mechanical manipulation and tissue strain exhibited by the VFs in isolation, analysis of voicing type information could complement existing vocal dose metrics as a surrogate of VF contact area and open quotient in voice use quantification [36]. One other plausible application is to integrate machine learning algorithms with existing clinical practices for the evaluation of voice quality and function, creating a hybrid expert system of voice assessment by incorporating other factors, such as subjective reports on vocal conditions. Such an expert system will help further understand the mechanisms of VF injury and repair and assist voice pathology medical diagnostics.

One study limitation is that diphthongs and voiced consonants were excluded from the analysis. They should be accounted for as additional voiced speech sounds in English. A comprehensive speech analysis is not possible unless an investigation of diphthongs and voiced consonants is completed. Future work should include the application of machine learning techniques to identify voice types in sentences and running speech and the investigation of additional voice types (such as falsetto). It would be of interest to investigate participants with contrastable severities of dysphonia and subjects with pathologies that contribute to specific variation of vocal fold mobility and vibration (nodules, unilateral paralysis, etc.). The NSA may offer clinicians an auxiliary tool of tracking voice type variation in long-term voice monitoring. The quantification of severity based on NSA signals should also be considered. The long-term goal of this research is to use the NSA to monitor vocal conditions for professional voice users and individuals suffering from disordered voice.

## Figures and Tables

**Table 1.** Confusion matrix and overall accuracy in voice type classification using a single feature.

Feature		PM	PB	PP	Overall Accuracy
*H*_1_	**TM**	35%	31%	34%	64.7%
	**TB**	12%	84%	4%	
	**TP**	26%	6%	68%	
*H*_2_	**TM**	35%	37%	28%	61.7%
	**TB**	12%	82%	7%	
	**TP**	29%	10%	61%	
*H*_3_	**TM**	24%	35%	41%	59.3%
	**TB**	7%	92%	2%	
	**TP**	17%	33%	50%	
*H*_4_	**TM**	2%	74%	24%	44.7%
	**TB**	2%	87%	11%	
	**TP**	1%	71%	28%	
SE	**TM**	0%	88%	12%	54.1%
	**TB**	0%	93%	7%	
	**TP**	0%	46%	54%	
*J*_*r*_	**TM**	54%	46%	0%	47.4%
	**TB**	25%	75%	0%	
	**TP**	42%	58%	0%	
*S*_*r*_	**TM**	92%	2%	6%	60.6%
	**TB**	18%	72%	10%	
	**TP**	56%	33%	11%	

TM: True Modal, TB: True Breathy, TP: True Pressed; PM: Predicted Modal, PB: Predicted Breathy, PP: Predicted Pressed.

**Table 2.** Overall classification accuracies (%) for the full set and the LOFO subsets.

	DT	LD	SVM	KNN	NN	Per-Set
Full Set	77.4	78.3	81.3	81.0	82.5	80.1 ± 2.1
−*H*_1_	74.9	78.4	82.1	81.7	83.3	80.1 ± 3.4
−*H*_2_	74.4	79.0	82.0	81.7	83.3	80.1 ± 3.5
−*H*_3_	75.4	78.0	81.5	79.9	82.7	79.5 ± 2.9
−*H*_4_	77.6	78.0	81.5	81.5	82.5	80.2 ± 2.3
-SE	74.4	74.3	78.3	78.5	77.9	76.7 ± 2.1
−*Jr*	75.4	76.8	81.1	80.1	83.3	79.3 ± 3.2
−*S*_*r*_	71.8	74.9	73.2	75.3	76.8	74.4 ± 1.9
Per-Classifier	75.2 ± 1.8	77.3 ± 2.5	80.1 ± 2.9	79.9 ± 2.2	81.5 ± 2.6	

**Table 3.** True Positive Rate (TPR), False Positive Rate (FPR) and Area Under the receiver operating characteristic Curve (AUC) of different classifiers for different voice types based on the full feature set.

		DT	LD	SVM	KNN	NN
Modal	TPR	0.73	0.70	0.79	0.77	0.81
	FPR	0.14	0.13	0.11	0.11	0.10
	AUC	0.85	0.89	0.92	0.92	0.93
Breathy	TPR	0.84	0.87	0.89	0.89	0.90
	FPR	0.10	0.10	0.09	0.09	0.08
	AUC	0.89	0.95	0.96	0.96	0.97
Pressed	TPR	0.69	0.75	0.74	0.75	0.91
	FPR	0.10	0.10	0.08	0.08	0.16
	AUC	0.86	0.91	0.92	0.93	0.94

**Table 4.** Overall classification accuracies (%) for the full set, the spectral set and the stability set.

	DT	LD	SVM	KNN	NN	Per-Set
Full Set	77.4	78.3	81.3	81.0	82.5	80.1 ± 2.1
*H*_1–4_ + SE	73.5	75.2	74.9	75.1	76.7	75.1 ± 1.1
*J*_*r*_ + *S*_*r*_	59.8	60.0	59.0	57.1	61.5	59.5 ± 1.6

## Abbreviations

NSA: Neck Skin Accelerometer
SLP: Speech Language Pathologist
GRABAS: Grade of Roughness, Breathiness, Asthenia, Strain
CAPE-V: Consensus Auditory-Perceptual Evaluation of Voice
GIF: Glottal Inverse Filtering
MFDR: Maximum Flow Declination Rate
MFCC: Mel-Frequency Spectral Coefficients
CPP: Cepstral Peak Prominence
SVM: Support Vector Machine
GMM: Gaussian Mixture Modal
NN: Neural Network
DT: Decision Tree
KNN: K-Nearest Neighbours
LOFO: Left-One-Feature-Out
SAL: Surface Acceleration Level
SPL: Sound Pressure Level
PCB: Printing Circuit Board
LDV: Lase Doppler Velocimetry
ICC: Intra-class Correlation Coefficient
VAD: Vocal Activity Detection
SE: Spectral Entropy
LD: Linear Discriminant
TP: True Pressed
PP: Predicted Pressed
TB: True Breathy
PB: Predicted Breathy
TM: True Modal
PM: Predicted Modal
TPR: True Positive Rate
FPR: False Positive Rate
AUC: Area Under the receiver operating characteristic Curve
